# Identification of novel QTLs for grain fertility and associated traits to decipher poor grain filling of basal spikelets in dense panicle rice

**DOI:** 10.1038/s41598-021-93134-7

**Published:** 2021-06-30

**Authors:** Sudhanshu Sekhar, Jitendra Kumar, Soumya Mohanty, Niharika Mohanty, Rudraksh Shovan Panda, Swagatika Das, Birendra Prasad Shaw, Lambodar Behera

**Affiliations:** 1 Crop Improvement Division, ICAR-National Rice Research Institute (NRRI), Cuttack, Odisha 753006 India; 2 Institute of Life Sciences, Bhubaneswar, Odisha 751023 India

**Keywords:** Genetics, Molecular biology, Plant sciences

## Abstract

High grain number is positively correlated with grain yield in rice, but it is compromised because of poor filling of basal spikelets in dense panicle bearing numerous spikelets. The phenomenon that turns the basal spikelets of compact panicle sterile in rice is largely unknown. In order to understand the factor(s) that possibly determines such spikelet sterility in compact panicle cultivars, QTLs and candidate genes were identified for spikelet fertility and associated traits like panicle compactness, and ethylene production that significantly influences the grain filling using recombinant inbred lines developed from a cross between *indica* rice cultivars, PDK Shriram (compact, high spikelet number) and Heera (lax, low spikelet number). Novel QTLs, *qSFP1.1*, *qSFP3.1*, and *qSFP6.1* for spikelet fertility percentage; *qIGS3.2* and *qIGS4.1* for panicle compactness; and *qETH1.2*, *qETH3.1*, and *qETH4.1* for ethylene production were consistently identified in both *kharif* seasons of 2017 and 2018. The comparative expression analysis of candidate genes like *ERF3*, *AP2*-like ethylene-responsive transcription factor, *EREBP*, *GBSS1*, E3 ubiquitin-protein ligase *GW2*, and LRR receptor-like serine/threonine-protein kinase *ERL1* associated with identified QTLs revealed their role in poor grain filling of basal spikelets in a dense panicle. These candidate genes thus could be important for improving grain filling in compact-panicle rice cultivars through biotechnological interventions.

## Introduction

Rice is a staple crop for about half of human beings all over the world, especially in Asia and Africa. However, since the last decade, the production of rice has remained more or less static to approximately 450 metric tons, which need to be increased at least 1.5 times by the year 2050 to feed the increasing world population^1^. To increase yield further, the breeding efforts have expanded the yield sink capacity of rice varieties mainly by increasing the number of spikelets per panicle^2^. As a result, rice varieties with large panicle bearing numerous spikelets have been successfully developed, like New plant type (NPT) lines by International Rice Research Institute (IRRI) and ‘super’ rice or ‘super’ hybrid rice in China^3,4^. However, poor grain filling particularly of the basal spikelets is the major cause of harnessing the high grain yield potential of these varieties^5^.

The anthesis in a rice panicle is not synchronous it is basipetal asynchronous^6^. Anthesis starts from the spikelets borne on the primary branches of the apical region of the panicle and proceeds towards the spikelets located on the basal primary or secondary branches in six to seven days. The asynchronous anthesis is largely responsible for variations in quality and weight of the kernels produced in the apical and basal spikelets with the basal spikelets producing smaller grains compared with that produced by the apical spikelets^7–9^. The difference in the quality of the grain produced by the apical and basal spikelets because of asynchronous anthesis is visible more in the compact panicle bearing numerous spikelets compared with that in the lax panicle bearing fewer (< 250) spikelets^9^. Many reasons have been indicated for differential grain filling in the apical and basal spikelets of compact panicle rice. Some of the earlier studies suggested comparatively lesser translocation of assimilates from the leaf sheath to the sink in the basal spikelets than that in the apical spikelets to be the cause of poor grain filling in the former than in the latter^7,10^. In contrast, it has been seen experimentally that poor grain filling in the basal spikelets of the compact-panicle is a result of sink limitation rather than the limitation of the flow of carbohydrate from the source^4^. Studies have shown that the assimilates partitioned into the endosperm cells of the basal spikelets, particularly of those located on the secondary branches of the panicle remain unused during the course of grain development^11,12^. Furthermore, spikelet thinning treatment in which sufficient numbers of apical spikelets are removed from the panicle just before heading has shown that the basal spikelets develop into a well-filled grain similar to that of the apical spikelets, suggesting that the basal spikelets of the compact panicle are genetically competent to produce well developed mature grain, the quality that could be masked by factors unknown so far^13^. The research conducted so far has revealed that the poor filling of grain in the basal spikelets compared with apical ones could be the low activity of the starch synthesizing enzymes, like sucrose synthase (SUS), starch synthase (SS), granule bound starch synthase (GBSS), UDPase, adenosine diphosphate glucose pyrophosphorylase (AGPase), starch debranching enzymes (DBE) and starch branching enzyme (SBE)^14–17^, although the evidences are only circumstantial. Further, sucrose transporter genes *OsSUT1*, *OsSUT2*, *OsSUT3*, *OsSUT4*, and *OsSUT5*^18,19^ and cell wall invertase (CIN) gene family *OsCIN1*, *OsCIN2*, *OsCIN4*, and *OsCIN7*^20^ have been reported a strong effect on grain filling. Grain Incomplete Filling1 (*GIF1*), a cell wall invertase (*OsCIN2*) regulates sucrose transport and unloading^16^ and *GIF2* an ADP-Glc pyrophosphorylase large subunit^21^, have roles in the regulation of grain filling during the early grain-filling stage in rice. In addition, comparative proteomics of apical and basal spikelets during early grain-filling stages in heavy panicled rice varieties has shown that endosperm cell division in inferior spikelets is lower than that of superior spikelets leads to poor grain filling in basal spikelets^22^. Besides, the plant hormone ethylene has been indicated to be involved in various regulatory and signalling pathways linked to the grain filling process and has an inhibitory role in grain filling^23^. The inhibitory role of ethylene in grain filling is basically indicated from the fact that the basal spikelets of compact panicle produce more ethylene than that of the lax- panicle, and ethylene receptors and the downstream signaling components of ethylene are constitutively expressed more in the compact panicle spikelets than that in the lax panicle spikelets^23^. Fu and Xue^24^ and Sekhar et al.^23^ have further proved that expression of RSR1, a rice AP2/EREBP family transcription factor (in the ethylene signaling pathway) is negatively correlated with the expression of type I starch synthesis like GBSS1 genes. High ethylene evolution in developing rice kernels suppresses the expression of most of the starch-synthesis genes that inhibits the activities of starch synthesis-related enzymes, leads to a poor grain-filling rate particularly in dense panicle basal spikelets^8,25^. Also, ethylene inhibitor (1-MCP) improves the dry matter partitioning and the development of basal spikelets by enhancing the expression of genes involved in starch-synthesizing enzymes and endosperm cell-cycle regulators in a dense-panicle rice cultivar^17^. An ethylene receptor, Ethylene response2 (ETR2), delays flowering that causes starch accumulation in stems and not translocated to grains, leading to low grain weight in rice^23,26^. All these studies suggested that grain filling or spikelet fertility is regulated by complex mechanisms and involved various gene products and hormonal regulation that determined grain yield. However, the factor responsible for poor grain filling in basal spikelets in dense panicled rice cultivars has yet to be elucidated.

Keeping in view the fact that the normal agricultural practices being followed and continuing conventional breeding programs are no more effective in increasing grain yield of rice, it is highly necessary to understand the reason for the trade-off between spikelet numbers and grain filling in compact panicle to increase the rice production. The identification of quantitative trait loci (QTLs) associated with complex phenotypic traits provides an opportunity for the genetic manipulation and improvement of the traits of interest^27–29^. Several genes/alleles associated with good grain filling were found in *indica* rice cultivars^30^. Hence, consorted efforts should be made to identify more elite novel genes for grain fertility from different *indica* sources and pyramid into extra-heavy panicle type high-yielding rice varieties^30^ to improve the grain filling. Therefore, the identification of novel elite genes/QTLs associated with traits like grain fertility, compactness (inter grain space), and ethylene production would unmask the factors behind the poor grain filling of dense panicle basal spikelets. So, in the present study, an attempt was made to identify novel QTLs and candidate genes associated with spikelet fertility, panicle compactness (inter-grain space), and phytohormone ethylene production using RIL mapping population developed from the cross between two *indica* rice cultivars, PDK Shriram (compact panicle, high spikelet number per panicle) and Heera (lax panicle, low spikelet number per panicle) and further to investigate the possible cause of poor grain filling or sterility in basal spikelets of the dense panicle.

## Results

### Phenological variation in the parents and RIL mapping population

**Figure 1:**
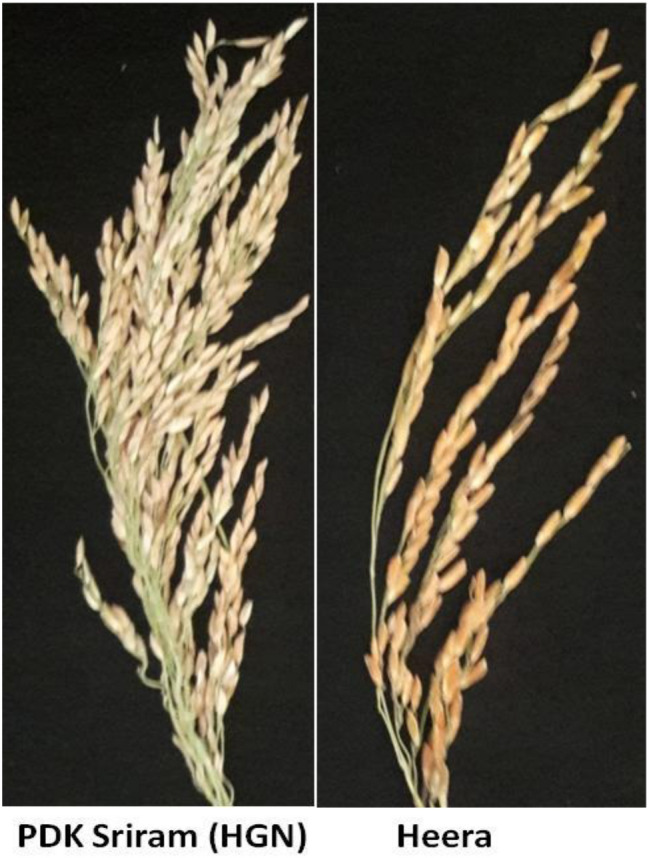
Parental rice cultivars PDK Shriram (HGN) and Heera showing compact- and lax-panicle type, respectively.

**Figure 2:**
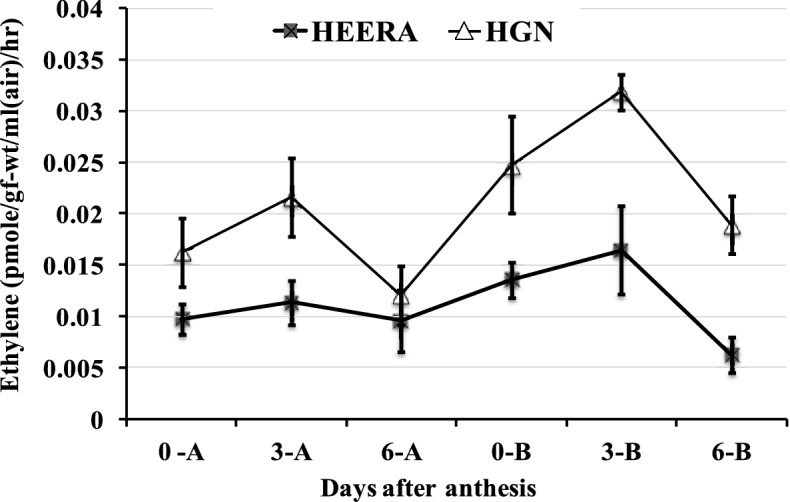
Ethylene production in apical and basal spikelets of compact panicled cultivar PDK Shriram (HGN) and lax-panicled cultivar Heera on different days after anthesis (0, 3 and 6). The data are the mean (± SD) of five observations. There was significant difference (p ≤ 0.05) for the ethylene production between apical and basal spikelets in HGN. A denotes apical spikelets; B denotes, basal spikelets.

The total spikelet number per panicle was much higher in PDK Shriram (350–360) than Heera (100–110) (Table 1, Fig. 1). The inter-grain space (IGS) was much less in PDK Shriram (Mean: 0.268 cm) compared with Heera (Mean: 0.661 cm). In contrast to SFP and IGS, the ethylene production was more in PDK Shriram in basal spikelets (Mean: 0.0319 pmol g^−1^ frwt ml^−1^ air h^−1)^ than in Heera (Mean: 0.0165 pmol g^−1^ frwt ml^−1^ air h^−1^) for 3 day after anthesis (Fig. 2). Furthermore, the production of ethylene was always more in the basal spikelets compared with the apical spikelets in all the days after fertilization in both the cultivars (Fig. 2). The phenological data for parents and two RIL lines 166A and 14A were shown in Table 1 for both the *kharif* seasons of 2017 and 2018. The spikelet fertility among RILs varied from 21.22 to 89.76% with an average of 69.23% in *kharif* 2017, while 45.22–97.13% with an average of 73.17% in *kharif* 2018 (supplementary Table S1). The IGS in the RIL population varied from 0.28 to 0.95 cm with an average of 0.51 cm in *kharif* 2017, while 0.26 to 1.03 cm with an average of 0.53 cm in *kharif* 2018. The ethylene production varied from 0.01 pmol g^−1^ frwt ml^−1^ air h^−1^ to 0.201 pmol g^−1^ frwt ml^−1^ air h^−1^ in *kharif* 2017, while 0.01 pmol g^−1^ frwt ml^−1^ air h^−1^ to 0.25 pmol g^−1^ frwt ml^−1^ air h^−1^ with an average of 0.0294 pmol g^−1^ frwt ml^−1^ air h^−1^ in *kharif* 2017 and 0.03 pmol g^−1^ frwt ml^−1^ air h^−1^ in *kharif* 2018 (Supplementary Table S1).

**Table 1:** Morphological feature of panicles of parental cultivars and two RILs selected based on high spikelet number (compact) and low grain number (lax) panicle and their fertility percentage.

Cultivars	Duration of cultivars (days)	Year	Panicle length (cm)	Total grain numbers	Filled grain (%)	Inter-grain space (cm)
HGN	140	2017	21.13 ± 0.54	350 ± 10	69.975	0.266 ± 0.06
		2018	22.14 ± 0.60	344 ± 11	70.235	0.270 ± 0.08
		Mean (2017 & 2018)	21.64 ± 0.58	347 ± 15	70.11	0.268 ± 0.07
Heera	90	2017	20.67 ± 1.13	100 ± 11	85.82	0.621 ± 0.03
		2018	20.96 ± 0.83	98 ± 10	87.41	0.701 ± 0.06
		Mean (2017 & 2018)	20.82 ± 0.48	99 ± 11	86.62	0.661 ± 0.04
RIL-166A	145	2017	21.82 ± 0.38	356 ± 12	59.42	0.286 ± 0.012
		2018	22.58 ± 0.31	361 ± 9	55.69	0.226 ± 0.071
		Mean (2017 & 2018)	22.2 ± 0.35	358 ± 10	57.56	0.256 ± 0.042
RIL-14A	88	2017	20.82 ± 0.51	98 ± 8	86.86	0.630 ± 0.081
		2018	20.11 ± 0.22	95 ± 10	88.49	0.690 ± 0.044
		Mean (2017 & 2018)	20.47 ± 0.36	96 ± 11	87.68	0.67 ± 0.064

Inter-grain space = Total length of primary branch / total number of spikelets per panicle. All values are mean of five replicates.

It was also observed the difference in the production of ethylene was higher between the basal spikelets of the two cultivars than between the apical spikelets with PDK Shriram showing more production of ethylene than Heera. In addition, the production of ethylene increased greatly in the basal spikelets in PDK Shriram with an increase in the days after anthesis from 0 to 3 days, but the increase was not much in Heera (Fig. 2). Supplementary Table S1 shows the values for skewness and kurtosis for SPF, IGS, and ethylene production for the *kharif* seasons of 2017 and 2018. The normal frequency distribution of RILs was observed in SPF and IGS (Supplementary Fig. 1a, 1b). The ethylene production recorded for 3 DAA in the basal spikelets of RILs, showed highly positive skewness and kurtosis (Supplementary Table S1).

### Correlation analysis between traits

Inter-grain space (IGS) showed a significant positive correlation with spikelet fertility percentage (SFP) for the seasons’ data, *kharif* 2017 (0.313) and *kharif* 2018 (0.325) at the level p ≤ 0.01 (Table 3). IGS, on the other hand, showed a significant negative correlation (− 0.128 in *kharif* 2017 and—0.191 in *kharif* 2018) with ethylene production (ETH) at an early stage after anthesis (3 DAA) in the RIL population at the level p ≤ 0.05. However, no significant correlation was observed between ETH and SFP in both the *kharif* seasons (Table 2).

**Table 2:** Correlation among spikelet fertility percentage (SFP), inter-grain space (IGS) and ethylene production (ETH) in 188 RILs developed from the cross between PDK Shriram (HGN) and Heera grown during *kharif* seasons of 2017 and 2018.

Pearson correlation						
	SFP		IGS		ETH	
	2017	2018	2017	2018	2017	**2018**
SFP	1	1	0.313**	0.325**	0.015	− 0.105
IGS	0.313**	0.325**	1	1	− 0.128*	− 0.191*
ETH	0.015	− 0.105	− 0.128*	− 0.191*	1	1
N	188					

**Correlation is significant at the 0.01 level (1-tailed).

*Correlation is significant at the 0.05 level (1-tailed).

### Identification of QTLs for spikelet fertility percentage, inter-grain space, and ethylene production

**Figure 3:**
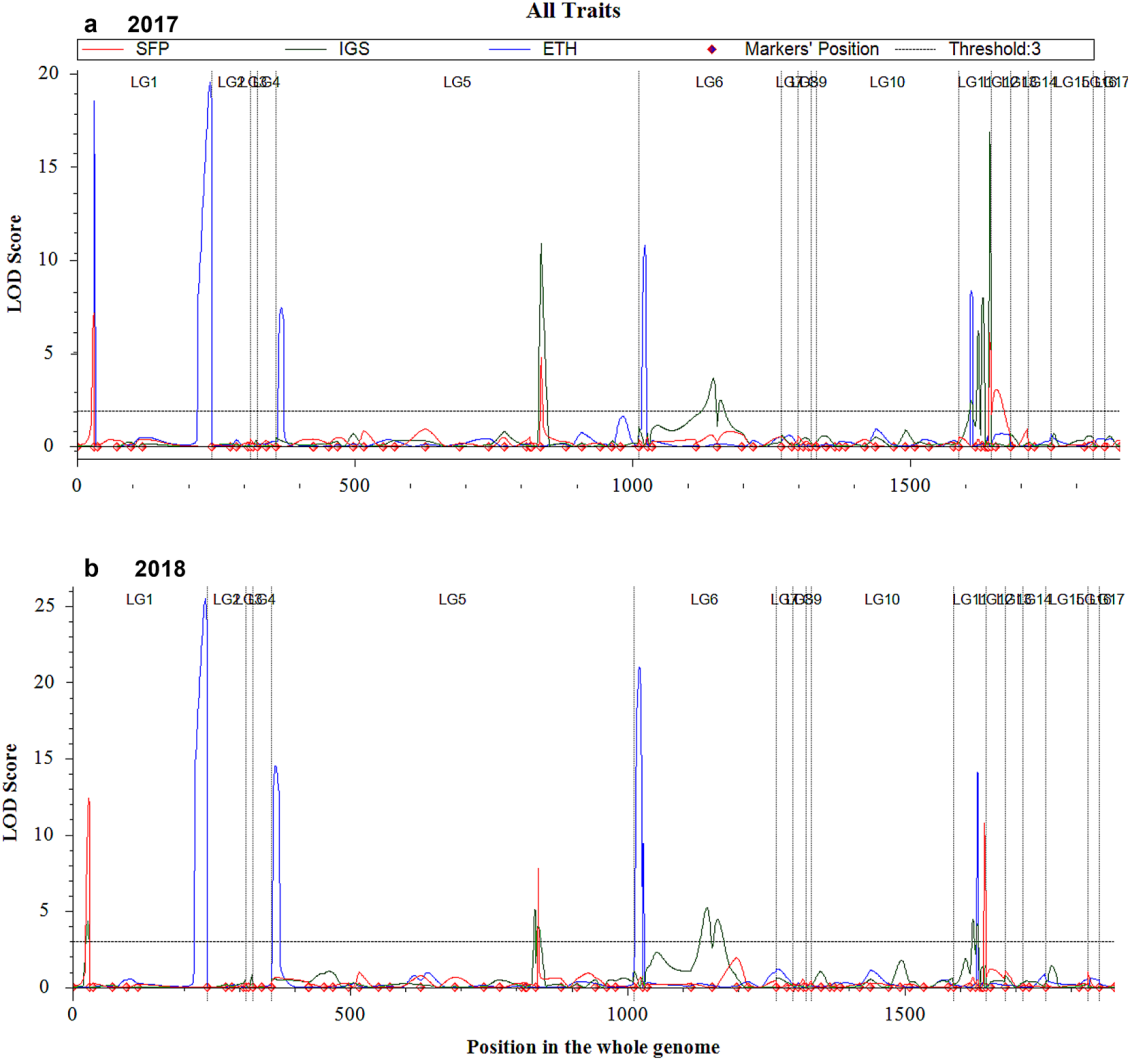
One-dimensional scanning of whole genome for identification of QTLs associated with spikelet fertility percentage (SFP), inter-grain space (IGS) and ethylene production (ETH) in *kharif* 2017 and *kharif* 2018, detected at threshold LOD score 3 using QTL ICI mapping V4.1 software. Graph shows identified QTL peaks on linkage groups. Fourteen peaks corresponding to fourteen QTLs were identified in *kharif* 2017 on chromosome 1 (LG1), chromosome 3 (LG5), chromosome 4 (LG6), chromosome 6 (LG11) and chromosome 8 (LG12). Fifteen peaks corresponding to fifteen QTLs were identified in *kharif* 2018 on chromosome 1 (LG1), chromosome 3 (LG5), chromosome 4 (LG6) and chromosome 6 (LG11). LG—Linkage group.

**Figure 4:**
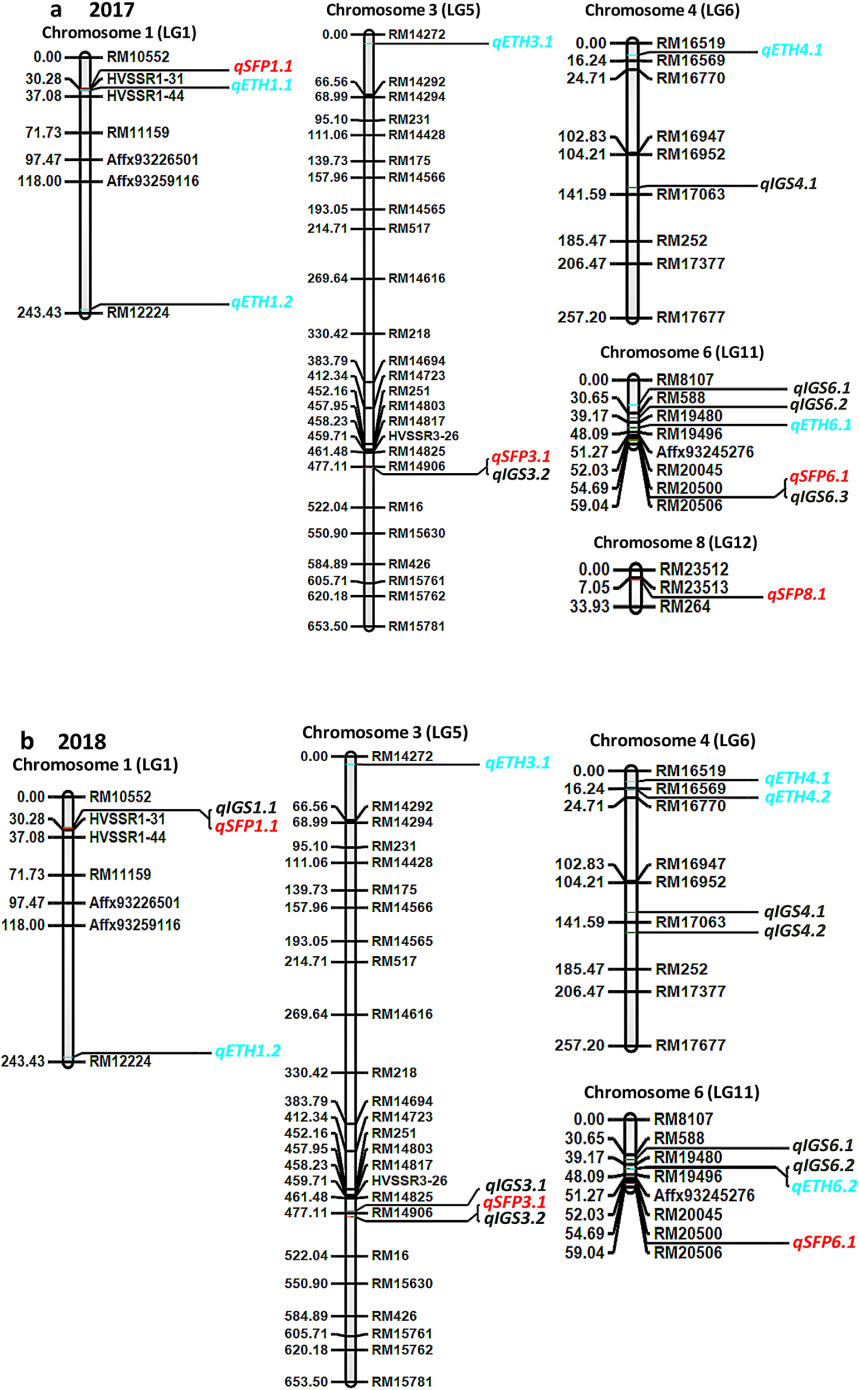
Linkage map showing QTLs associated with spikelet fertility (SFP), inter-grain space (IGS) and ethylene production (ETH). QTL identification was done using integrated QTL IciMapping, Version 4.1 software for (**a**) *kharif* 2017, and (**b**) *kharif* 2018. Markers and QTLs are represented on right side of linkage group, while values on left side represented linkage distance in cM, LG-Linkage group.

A total of 118 (9.83%) out of 1200 SSR markers and 22 (9.78%) out of 225 SNP markers (Supplementary Table S2, S3) were found polymorphic between parents, PDK Shriram and Heera. Seven markers that showed highly segregation distortion (P < 0.0001) were not used in linkage map construction. The remaining 133 polymorphic markers were used to construct a linkage map. A total of 17 linkage groups were constructed after screening with LOD threshold 3 using the IciMapping V4.1 software. The linkage map covered a total genetic distance of 1878.3 cM (Supplementary Table S5) with an average marker interval of 19.17 cM. One-dimensional scanning of the whole genome was carried out with mapping parameters of step size 1 cM with PIN (probability value for entering variables) value 0.001 and a threshold LOD score of 3 to identify significant QTLs associated with spikelet fertility percentage (SFP), inter grain space (IGS) and ethylene production (ETH) in *kharif* seasons of 2017 and 2018 (Fig. 3). A total of 14 QTLs was identified in *kharif* 2017, while 15 QTLs were identified in *kharif* 2018 on five different chromosomes of rice (Table 3). A total of four QTLs (*qSFP1.1*, *qSFP3.1*, *qSFP6.1*, and *qSFP8.1*) for spikelet fertility percentage, eight QTLs (*qIGS1.1*, *qIGS3.1*, *qIGS3.2*, *qIGS4.1*, *qIGS4.2*, *qIGS6.1*, *qIGS6.2*, and *qIGS6.3*) for inter-grain space, and seven QTLs (*qETH1.1*, *qETH1.2*, *qETH3.1*, *qETH4.1*, *qETH4.2*, *qETH6.1*, and *qETH6.2*) for ethylene production were identified. Four QTLs (*qSFP1.1*, *qSFP3.1*, *qSFP6.1*, and *qSFP8.1*) for spikelet fertility percentage, five QTLs (*qIGS3.2*, *qIGS4.1*, *qIGS6.1*, *qIGS6.2*, and *qIGS6.3*) for inter-grain space and five QTLs (*qETH1.1*, *qETH1.2*, *qETH3.1*, *qETH4.1*, and *qETH6.1*) for ethylene production were identified in *kharif* 2017 (Table 3, Fig. 4a), while three QTLs (*qSFP1.1*, *qSFP3.1* and *qSFP6.1*) for spikelet fertility percentage, seven QTLs (*qIGS1.1*, *qIGS3.1*, *qIGS3.2*, *qIGS4.1*, *qIGS4.2*, *qIGS6.1*, and *qIGS6.2*) for inter-grain space and five QTLs (*qETH1.2*, *qETH3.1*, *qETH4.1*, *qETH4.1*, and *qETH6.2*) for ethylene production were identified in *kharif* 2018 (Table 3, Fig. 4b). Three QTLs (*qSFP1.1*, *qSFP3.1* and *qSFP6.1*) for spikelet fertility percentage, four QTLs (*qIGS3.2*, *qIGS4.1*, *qIGS6.1* and *qIGS6.2*) for inter-grain space and three QTLs (*qETH1.2*, *qETH3.1*, and *qETH4.1*) for ethylene production were consistently identified in both *kharif* seasons of 2017 and 2018 (Table 3, Fig. 4). QTLs explained phenotypic variance, which varied from 5.423% (*qETH4.1* in kharif 2017) to 12.653% (*qIGS6.1* in *kharif* 2017) (Table 3). The LOD score found to vary from 3.045 on chromosome 8 (*qSFP8.1* in *kharif* 2017) to 25.462 on chromosome 6 (*qETH1.1* in *kharif* 2018). The QTLs for spikelet fertility percentage and inter-grain space on 6 (*qSFP6.1*, *qIGS6.3*) were found to be same region on chromosome 6 between RM20500 and RM20506 in *kharif* 2017, while QTL (*qSFP1.1*, *qIGS1.1*) found to be same region on chromosome 1 between RM10552 and HVSSR1-31 in *kharif* 2018. Further, *qSFP3.1* and *qIGS3.2* were also identified in the same region between RM14906 and RM16 on chromosome 3 in both *kharif* seasons of 2017 and 2018, while *qIGS6.2* and *qETH6.2* were identified in the same region between RM19480 and RM20045 in *kharif* season of 2018 (Table 3, Fig. 4).

**Table 3:** QTLs identified for spikelet fertility percentage (SFP), inter-grain space (IGS) and ethylene production (ETH) using RILs derived from PDK Shriram (HGN) and Heera in *kharif* seasons of 2017 and 2018.

Year	Traits name	QTL name	LG	Chr#	Position (cM)	Left marker	Right marker	LOD	PVE (%)	Add	Contributor allele (HGN/Heera)
2017	SFP	*qSFP1.1*	1	1	30	RM10552	HVSSR1-31	7.037	10.636	− 23.856	Heera
		*qSFP3.1*	5	3	478	RM14906	RM16	4.752	10.23	− 23.826	Heera
		*qSFP6.1*	11	6	55	RM20500	RM20506	6.107	9.995	16.229	HGN
		*qSFP8.1*	12	8	9	RM23513	RM264	3.045	7.567	− 2.997	Heera
	IGS	*qIGS3.2*	5	3	478	RM14906	RM16	10.874	11.464	− 0.246	Heera
		*qIGS4.1*	6	4	135	RM16952	RM17063	3.656	11.442	− 0.052	Heera
		*qIGS6.1*	11	6	35	RM588	RM19480	6.191	12.653	0.153	HGN
		*qIGS6.2*	11	6	44	RM19480	RM19496	7.983	11.831	0.153	HGN
		*qIGS6.3*	11	6	56	RM20500	RM20506	16.854	9.093	0.246	HGN
	ETH	*qETH1.1*	1	1	31	HVSSR1-31	HVSSR1-44	18.530	5.714	− 0.045	Heera
		*qETH1.2*	1	1	240	Affx93259116	RM12224	19.556	5.996	− 0.045	Heera
		*qETH3.1*	5	3	10	RM14272	RM14292	7.458	6.469	0.035	HGN
		*qETH4.1*	6	4	11	RM16519	RM16569	10.794	5.423	0.048	HGN
		*qETH6.1*	11	6	23	RM19496	RM20045	8.35	6.447	0.043	HGN
2018	SFP	*qSFP1.1*	1	1	29	RM10552	HVSSR1-31	12.375	9.792	− 24.957	Heera
		*qSFP3.1*	5	3	481	RM14906	RM16	7.812	9.528	− 24.956	Heera
		*qSFP6.1*	11	6	56	RM20500	RM20506	10.759	10.353	27.996	HGN
	IGS	*qIGS1.1*	1	1	28	RM10552	HVSSR1-31	4.343	9.555	− 0.157	Heera
		*qIGS3.1*	5	3	475	RM14825	RM14906	5.103	10.487	− 0.126	Heera
		*qIGS3.2*	5	3	481	RM14906	RM16	4.094	10.999	− 0.135	Heera
		*qIGS4.1*	6	4	132	RM16952	RM17063	5.204	11.47	− 0.082	Heera
		*qIGS4.2*	6	4	151	RM17063	RM252	4.451	9.037	− 0.080	Heera
		*qIGS6.1*	11	6	35	RM588	RM19480	4.457	5.984	0.131	HGN
		*qIGS6.2*	11	6	43	RM19480	RM19496	4.175	6.35	0.131	HGN
	ETH	*qETH1.2*	1	1	239	Affx93259116	RM12224	25.462	5.471	− 0.051	Heera
		*qETH3.1*	5	3	8	RM14272	RM14292	14.527	6.873	0.047	HGN
		*qETH4.1*	6	4	10	RM16519	RM16569	21.001	5.445	0.048	HGN
		*qETH4.2*	6	4	17	RM16569	RM16770	9.416	6.561	0.048	HGN
		*qETH6.2*	11	6	43	RM19480	RM19496	14.112	6.846	0.052	HGN

LG-Linkage group; Chr#—Chromosome number, PVE-phenotypic variance.

### Analysis of candidate genes associated with identified QTLs

In silico search was done to identify genes present in the chromosomal regions associated with identified QTLs. NCBI gene bank database (https://www.ncbi.nlm.nih.gov/) and rice genome annotation project (RAP-DB) were used to search genes in QTL regions. 1438, 1929 and 111 genes were identified in QTL regions, Affx93259116-RM12224 (*qETH1.2*), RM588-RM20045 (*qIGS6.1*, *qIGS6.1*, and *qETH6.1*), and RM20500-RM2050 *(qSFP6.1*, and *qIGS6.3*), respectively (Supplementary Table S7a). From among these genes, important candidate genes in QTL regions were listed in Supplementary Table S7b, c, d. Six genes were predicted for ethylene production in the QTL region of *qETH1.2* on chromosome 1 (Supplementary Table S7b). On chromosome six, ten genes were predicted in the QTL region of *qIGS6.1*, *qIGS6.2*, and *qETH6.1* for inter-grain space and ethylene production (Supplementary Table S7c), while 5 genes were predicted in the QTL region of *qSFP6.1* and *qIGS6.3* associated with spikelet fertility percentage and inter-grain space, respectively (Supplementary Table S7d).

### Expression analysis of candidate genes associated with identified QTLs

Comparative expression studies of seven candidate genes associated with three identified QTL regions containing six QTLs (*qETH1.2*, *qIGS6.1*, *qIGS6.2 and qETH6.1*, *qSFP6.1* and *qIGS6.3*) were carried out at the exponential stage of grain filling at 6 days, 9 days, and 12 days after anthesis in both apical and basal spikelets of parents (PDK Shriram and Heera) and two RILs, one lax-panicle low grain (RIL-14A) and one compact-panicle high grain number (RIL-166A). One ethylene-responsive transcription factor 8 also named as ethylene-responsive factor 3 (ERF3) associated with *qETH1.2* on chromosome 1 expression was higher in basal spikelets as compared to apical spikelets in compact panicle PDK Shriram and RIL-166A, while their expression was not significant between apical and basal spikelets in lax panicle Heera and RIL-14A (Supplementary Fig. 2a). Further, five candidate genes identified on chromosome 6 in the QTL region of *qETH6.1*, *qIGS6.1*, and *qIGS6.2* were selected for their expression analysis. Two ethylene-responsive genes, AP2-like ethylene-responsive transcription factor (Supplementary Fig. 2b) and EREBP (Fig. 2c) identified in the region of QTL, *qETR6.1* showed higher expression in basal spikelets of compact panicle cultivar, PDK Shriram and RIL-166A, while no significant difference was found the expression in apical and basal spikelets of lax panicle cultivar, Heera and RIL-14A. The expression pattern of GBSS1 (Supplementary Fig. 2d) identified in the QTL region of *qIGS6.1* and *qIGS6.2* shows opposite to the expression pattern of the other genes (Supplementary Fig. 2). The enzyme showed a greater expression in the apical spikelets compared with the basal spikelets in PDK Shriram and RIL-166A on all the days of post-anthesis (Supplementary Fig. 2b). In Heera and RIL-14A, the expression of the enzyme was more or less similar in both apical and basal spikelets (Supplementary Fig. 2d). E3 ubiquitin-protein ligase (GW2)(LOC107276853), which was linked to the QTL, *qSFP6.1*, and *qIGS6.3* in the marker region RM20500**-**RM20506 on chromosome 6 showed higher expression in the basal spikelets compared with the apical spikelets in the compact panicle cultivar, PDK Shriram and RIL-166A (Supplementary Fig. 3a). However, the opposite was the case with regard to the expression of E3 ubiquitin-protein ligase in the lax panicle Heera and RIL-14A sample (Supplementary Fig. 3a). The expression pattern of serine carboxypeptidase II-2 (LOC4340344), which was also identified on chromosome 6 in the marker region RM19480- RM19496 of QTL *qIGS6.2* was similar to E3 ubiquitin-protein ligase. The expression of the protein was higher in the basal spikelets compared with the apical ones in PDK Shriram (HGN) and RIL-166A, while the protein had a lower expression in the basal spikelets compared with the apical ones in Heera and RIL-14A on all the days post-anthesis, except on 6 DAA (Supplementary Fig. 3b). The expression pattern of the LRR receptor-like serine/ threonine-protein kinase ERL1 genes, which was also identified in the QTL region of *qIGS6.2* and *qETH6.1* on chromosome 6, was somewhat different from E3 ubiquitin-protein ligase and serine carboxypeptidase II-2. The expression of the protein was although mostly higher in the basal spikelets compared with the apical ones in general in PDK Shriram and RIL-166A, the expression was low in the basal spikelets than in the apical spikelets on 6 DAA in RIL-166A (Supplementary Fig. 3c). The expression of the protein on the other hand was much lower in the basal spikelets than in the apical spikelets in Heera, particularly on 9 and 12 DAA (Supplementary Fig. 3C). The expression of LRR receptor-like serine/threonine-protein kinase ERL1, however, remained more or less similar in both apical and basal spikelets in RIL-14A (Supplementary Fig. 3c), in contrast to the expression of E3 ubiquitin-protein ligase (Supplementary Fig. 3a) and serine carboxypeptidase II-2 (Supplementary Fig. 3b). Their expression was found upregulated in apical spikelets in Heera as compared to basal spikelets. However, in compact panicle cultivar PDK Shriram and RIL-166A, its expression was more in basal spikelets as compared to apical.

## Discussion

The grain filling stage is crucial and very complex in the rice life cycle. Several factors regulate the grain filling process determining the final grain yield. The number of spikelets per panicle plays an important role in determining the grain yield of the crop. Hence, increasing the spikelet number per panicle is essential to enhance the yield potential of rice. However, breeding programs to increase the number of spikelets per panicle is accompanied by poor filling of the basal spikelets particularly in the secondary branch that limits the grain yield and quality, as is evident from the poor grain filling percentage of PDK Shriram bearing numerous spikelets compared with the high grain filling percentage of Heera bearing fewer spikelets (Table 1). Similar findings have been noted in other studies as well ^2,5,6^. The major factor that has come to knowledge in the poor filling of grains on a panicle bearing numerous spikelets is a decrease in inter-grain space (IGS), which was also observed in the present study in PDK Shriram (Table 1), as well as reported by others^5,17,23^. In addition, higher production of ethylene in the basal spikelets of the compact panicle has also been reported to influence grain filling negatively ^8,17,23,25^, similar to that found in PDK Shriram (Fig. 2). However, the mechanistic details of inhibition of the grain filling process in basal spikelets of compact-panicle bearing numerous spikelets are yet to be understood. Nevertheless, in the present study, the identification of QTLs controlling the traits and their associate genes may unmask the cause of inhibition of grain filling in high spikelet number compact panicle cultivars at the genetic level.

The RIL population developed from PDK Shriram and Heera showed normal distribution for two phenotypic traits, SFP and IGS (Supplementary Fig. 1a, 2b, Supplementary Table S1) in both *kharif* seasons of 2017 and 2018. No significant skewness or kurtosis was found for these traits, while ethylene (ETH) production showed skewed distribution^31^. No significant correlation was found between ETH and SFP (Table 2), indicating that ethylene production might not be tightly linked with SFP, in contrast to reports available that high ethylene production in the spikelets is the cause of poor grain filling in basal spikelets of the compact panicle^8,23^. Rather, SFP might be linked to factors other than ETH, like IGS. Support to no possible link of SFP with ETH comes from the fact that in the present case, a lesser SFP was also observed in the RIL population showing low ethylene production (Supplementary Fig. 1). Nevertheless, the inhibitory role of ethylene may not be totally ruled out, as the ethylene-induced signalling component has been found to be inhibitory to granule bound starch synthase^23,24^. A significant positive correlation between SFP and IGS, is, however, certainly visible from the data of the RIL population (Table 2). It means inter-grain space (IGS) in panicle has an inhibitory role of grain filling that is lower the IGS, higher the poor grain filling particularly seen in basal spikelets. Wang et al.^32^ reported a negative relationship of poor grain filling with IGS. The compact panicle cultivars having lower inter-grain space showed lower grain weight and width, and higher chalky grain percentage and amylose content among grains within a panicle than the lax panicle cultivars^33^.

In our study, 118 (9.83%) out of 1200 SSR markers, and 22 (9.78%) out of 225 SNP markers showed polymorphism between parents, PDK Shriram (HGN) and Heera. These polymorphic markers were used to genotype 188 RILs of the cross between PDK Shriram (HGN) and Heera. Among these polymorphic markers, seven SSR markers showed highly segregation distortion (P < 0.00001) (Supplementary Table S4). These seven SSR markers were not considered for linkage mapping and QTL analysis because segregation distortion (SD) significantly affects linkage map construction and identification of QTLs^34^. Segregation distortion can be caused by competition among gametes for preferential fertilization and by abortion of the male or female gametes or zygotes at some stage of the development cycle^34,35^. SD can also occur as a result of conscious or unconscious selection during the development of mapping populations (while forwarding the materials over generations or while sampling). SD in permanent mapping populations such as RILs derived following the single-seed-decent method is due to the cumulative effect of both genetic and environmental factors on multiple generations^36–38^. Hence, it affects the accuracy of linkage map construction by introducing errors in map distance estimation, and marker order and thus could affect mapping quantitative trait loci (QTLs) when many SDs are present^35,39^.

A total of seven novel QTLs associated with ethylene production (*qETH1.1*, *qETH1.2*, *qETH3.1*, *qETH4.1*, *qETH4.2*, *qETH6.1*, and *qETH6.2*) were identified. No QTL has been identified earlier for ethylene production in these QTL regions. A total of four QTLs, *qSFP1.1*, *qSFP3.1*, *qSFP6.1*, and *qSFP8.1* for spikelet fertility percentage have been identified. Three QTLs, *qSFP1.1*, *qSFP3.1*, and *qSFP6.1* associated with spikelet fertility percentage are found to be novel as no QTL has been previously identified in regions between RM10552 and HVSSR1-31 on chromosome 1, RM14906, and RM16 on chromosome 3, and between RM20500 and RM20506 on chromosome 6, respectively. A total of seven QTLs (*qIGS1.1*, *qIGS3.1*, *qIGS3.2*, *qIGS4.1*, *qIGS4.2*, *qIGS6.1* and *qIGS6.2*) have been identified for inter-grain space. Further, *qIGS3.2* and *qIGS4.1* was novel as no QTL for panicle density or inter-grain space has been detected previously in the associated QTL region. Previously, QTL on chromosome 1 named *sf1* associated with spikelet fertility in the region of 34,264,553 bp–36,658,883 bp and on chromosome 6, QTL for spikelet density named *sd6* in the region of 6,023,974 bp–9,537,572 bp have been identified^40^. These QTLs are present in the marker region of detected QTLs *qIGS6.1* and *qIGS6.2*. A total of four QTLs have been identified for spikelet fertility previously, *qFER-6* in the region of 3,416,533 bp–3416728^41^, *qSPTF6* in the region of 4,234,080 bp–5,096,867 bp^42^, *S5* in the region of 6,283,432 bp–9284248 bp^43^, and *spf6* in the region of 6,283,432 bp-7177183 bp^44^. The traits, SFP, IGS, and ETH may have controlled with expression of certain genes associated with identified QTLs. The important to note was, however, that QTLs, *qSFP1.1*, *qIGS1.1* and *qSFP6.1*, *qIGS6.3* were identified in the same regions of chromosome 1 and chromosome 6, respectively (Table 3 and Fig. 4). Interestingly, these two traits also bear a significant correlation between them (Table 2), suggesting that the genes are associated with QTLs for SFP and IGS. Further, *qETH6.2* shared the same region with *qIGS6.2* on chromosome 6, suggesting that the inhibition in grain filling could be linked to the production of ethylene or inter-grain space at least some of the genes regulating IGS and ETH share a common region on the chromosome, and the genes associated with these QTLs may have pleiotropic effects on regulation of these traits.

**Figure 5:**
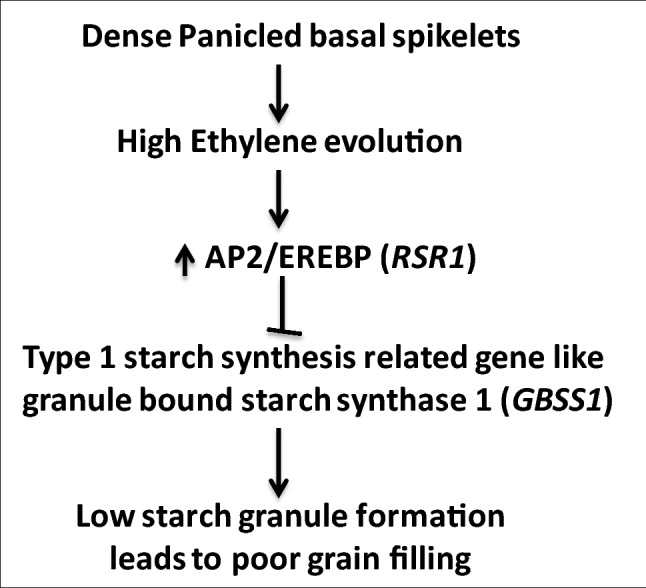
Model for poor grain filling in basal spikelets of dense panicle of rice, regulated by phytohormone ethylene*. AP2-EREBP* acts as a negative regulator of starch synthesis related genes leads to poor grain filling.

The apical spikelets were fully filled with good quality in dense panicled rice while in lax panicled (low grain number per panicle) rice cultivar, both apical and basal spikelets comparatively were good filled^5^. Hence, the comparative expression studies of identified candidate genes in apical and basal spikelets in both dense and lax-panicled rice cultivar PDK Shriram with RIL-166A and Heera with RIL-14A were done to know how their expression affects the grain filling in basal spikelets of dense panicled rice cultivar. The gene, *ERF3* (Os01g0797600), an ethylene-responsive downstream signaling component associated with QTL, *qETH1.2* on chromosome 1 shows higher expression (Supplementary Fig. 2a) in basal spikelets as compared to apical spikelets in compact panicle cultivar, while its expression was almost the same in apical and basal spikelets of lax panicle cultivar, Heera and RIL-14A, indicate that its expression was reciprocated the poor grain filling in basal spikelets in dense panicled rice cultivar. Similar results were also found for the expression of AP2-like ethylene-responsive transcription factor (Os06g0145700) (Supplementary Fig. 2b) and EREBP transcription factor (Os06g0194000) (Fig. 2c) associated with QTL, *qETH6.1* on chromosome 6. The results indicated that due to the higher evolution of ethylene in basal spikelets of compact panicle cultivar, the perception of ethylene might be higher that leads to higher expression of downstream ethylene signaling component in basal spikelets^23^. Further, AP2 like ethylene-responsive element-binding protein family transcription factor, also known as rice starch regulator (RSR1) and it negatively regulates the expression of type I starch synthesis genes^24^. Hence, due to higher expression of AP2 like EREBP family transcription factor in basal spikelets of compact panicle cultivar, there might be inhibition of expression of starch synthesis related genes may lead to poor grain filling in inferior spikelets. Further, type 1 starch synthesis-related gene, granule bound starch synthase 1 (*GBSS1*) was also identified in the QTL region of *qIGS6.1* and *qIGS6.2*. Their Spatio-temporal expression analysis of the enzyme reciprocated the grain filling pattern in the lax- as well as the compact-panicle, as the expression of the enzyme was more or less similar in both the apical and basal spikelets of the lax-panicle cultivar, Heera and RIL-14A, while the expression of the enzyme was downregulated in the basal spikelets compared with the apical ones in compact panicle cultivar, PDK Shriram and RIL-166A indicating the negative regulator of the expression of type I starch synthesis genes by AP2/EREBP family transcription factor. So, we propose a model for ethylene-mediated poor grain filling in basal spikelets of dense panicle rice cultivars (Fig. 5). Further, overexpression of AP2/ERF family transcription factor OsEATB (ERF protein associated with tillering and panicle branching was also reported to promote the branching and involve in reduction of panicle length through restricting internode elongation by down-regulating a GA biosynthetic gene ent-kaurene synthase A^45^ that leads to lower inter-grain space and increases compactness having numerous spikelets per panicle. Harrop et al.^46^ also reported that *AP2/EREBP*-like genes were involved in inflorescence branching and architecture in domesticated rice. E3 ubiquitin-protein ligase (*GW2*) has been reported to play a very important role in the regulation of weight of spikelets; the loss of function of *GW2* increases grain filling rate and larger spikelets hull^47^. Thus, a greater expression of this gene in the basal spikelets in the compact-panicle cultivar, PDK Shriram and RIL-166A (Supplementary Fig. 3a) might be one of the possible causes of poor filling of grains in them compared with the apical spikelets. Song et al.^47^ has also reported that *GW2* negatively regulates cell division by targeting its substrate(s) to proteasomes for regulated proteolysis. Furthermore, Choi et al.^48^ reported that *GW2* negatively regulates the seed size by targeting EXPLA1 for degradation through its E3 ubiquitin ligase activity. In addition, *GW2* regulates the seed size through direct interactions with proteins involved in carbohydrate metabolism by modulating their activity or stability and controlling disulfide bond formation in various proteins during seed development^49^. Since both the parent PDK Shriram and RIL-166A shows poor grain filling in the basal spikelets, and the same is accompanied by the compactness of their panicle, a low IGS in them could be a result of the pleiotropic effect of greater expression of E3 ubiquitin-protein ligase in the basal spikelets compared with apical, the possible cause of poor grain filling in the former than in the latter. The relationship between the spatial difference in expression of E3 ubiquitin-protein ligase and panicle compactness or panicle architecture, however, certainly needs further detailed investigation. Serine carboxypeptidase II-2 was another important enzyme that was identified in the QTL region of *qIGS6.2* and *qETH6.1* and found a greater expression in the basal spikelets compared with apical ones in PDK Shriram and RIL-166A (Supplementary Fig. 3b). The role of serine carboxypeptidase II-2 has so far not been investigated. However, some other serine carboxypeptidase like *GS5/OsSCP26* and *SCP46* has been reported to be a positive regulator of grain width and weight^50,51^. Higher expression of LRR receptor-like serine/threonine-protein kinase in the basal spikelets compared to the apical ones in PDK Shriram and RIL-166A (Supplementary Fig. 3c) could also be the reason for a low IGS and compactness of panicle in them in contrast to Heera and RIL-14A. The possible role of LRR receptor-like serine/threonine-protein kinase in regulating IGS in panicle is reflected from the fact that the enzyme is the largest receptor-like protein kinase having a diverse role in plant growth, development including organogenesis, morphogenesis, hormone signaling, and abiotic and biotic stress response in plants^52,53^. It has also been reported that LRR receptor-like serine/threonine-protein kinase ERL1 regulates inflorescence architecture and organ shape as well as stomatal patterning, including density and clustering, together with ER and ERL2^54–60^. Thus, the QTLs, *qIGS6.1*, *qIGS6.2*, and *qETH6.1* on chromosome 6 that harbour genes encoding proteins like LRR receptor-like serine/threonine-protein kinase remains to be answered how their higher expression in the basal spikelets in comparison with the apical spikelets would change the entire panicle architecture from lax to compact type.

## Conclusions

Grain filling in rice is a complex process, particularly keeping in view the differential filling of the grains in the spikelets based on their spatial location in compact panicles. The differential filling of grains in the compact panicle in which the basal spikelets remain largely unfilled or poorly filled has, in fact, been a major obstacle in rice breeding programs intended to increase rice production. An attempt was made to understand the molecular basis of differential grain filling through the identification of QTLs for spikelet fertility and its associated traits like inter-grain space, and ethylene production in the mapping RIL population of Heera (lax panicle) and PDK Shriram (compact panicle). Novel QTLs, *qSFP1.1*, *qSFP3.1*, and *qSFP6.1* for spikelet fertility *qIGS3.2* and *qIGS4.1* for inter-grain space and *qETH1.2*, *qETH3.1*, and *qETH4.1* for ethylene production are the useful QTLs that may have role in regulation of grain filling of basal spikelets in dense panicle rice. Five genes associated with these traits like ERF3, AP2-like ethylene-responsive transcription factor (Os06g0145700) and EREBP transcription factor (Os06g0194000), granular bound starch synthase 1 and E3 ubiquitin-protein ligase stand distinct are involved in the regulation of grain filling in basal spikelets of dense panicles, while the role of serine carboxypeptidase II-2, LRR receptor-like serine/threonine-protein kinase in compactness of panicle and grain filling was not known. These genes thus could be important candidate genes in improving the grain filling in compact-panicle rice cultivars showing poor grain filling through biotechnological interventions. The QTLs harbouring these genes may be useful to transfer the grain filling trait into the rice cultivars of interest through the molecular breeding approaches.

## Materials and methods

### Plant materials

Two *indica* rice cultivars, PDK Shriram (HGN) and Heera, contrast for spikelet numbers and panicle compactness were selected for the identification of QTLs associated with spikelet fertility percentage, panicle compactness (inter-grain spacing), and phytohormone ethylene production. PDK Shriram bears compact and heavy panicle with high spikelet numbers (350–360), while Heera bears lax-panicle with less number of spikelets (100–110) (Fig. 1). A mapping population consisting of 188 F_11_ recombinant inbred lines was developed from the cross PDK Shriram and Heera by single-seed descent method.

### Phenotypic evaluation of RIL mapping population for spikelet fertility and panicle compactness

All 188 RILs along with parents, PDK Shriram and Heera were planted in the experimental fields of ICAR-National Rice Research Institute, Cuttack, Odisha, India during the *kharif* seasons of 2017 and 2018 following an Alpha Lattice design each with two replications. The 25 days old seedlings were transplanted in the experimental plots. The row-to-row spacing of 20 cm and plant-to-plant spacing of 15 cm were maintained. Each row contained 20 single plants. The fertilizer dose of 80 kg N, 40 kg P_2_O_5,_ and 40 kg K_2_O_5_ per hectare was applied. The standard agronomic practices and need-based plant protection measures were undertaken for normal plant growth. Five plants were selected from the middle of the row from each replication and observations were recorded on the number of spikelets per panicle, number of sterile spikelets per panicle, percentage of filled spikelets/grains per panicle, panicle length, and length of the primary branches per panicle. The total length (cm) of the primary branches was divided by the total number of spikelets on the panicle to give the inter-grain space. Three panicles per plant for 5 plants with two replications were used for the generation of data on the panicle morphology. Mean data were used for further analysis.

### Estimation of ethylene production in the spikelets

Ethylene production in the apical and basal spikelets of the parents PDK Shriram and Heera was measured on the early days of anthesis, such as 0, 3, and 6 days after anthesis (DAA). The primary tillers were used to harvest for the collection of spikelets at various stages of anthesis. The differences in anthesis of spikelets on basal branches were 4–5 days after anthesis of apical spikelets and it was taken as 0 day for basal spikelets. The apical and basal spikelets sampled were moistened and immediately inserted separately in rubber stopper 10 ml tubes and sealed after 20 min. The sealed tubes with spikelets were incubated in darkness for 5 h. After incubation, the headspace gas (1 ml) was drawn with the help of airtight chromatography syringe and injected into the GC column of gas chromatography (Varian CP-3800) fitted with a flame ionization detector. The data were represented as the mean ± SD of five observations and expressed as pmol ethylene produced. h^−1^ g^−1^frwt ml^−1^ headspace air^23^. Ethylene production in all 188 RILs was also measured for 3 days after the anthesis of basal spikelets to generate phenological data for ethylene production.

### Genotyping of RIL mapping population

Genomic DNA isolation was carried out from the leaf tissues of the parents and all 188 RILs using the Cetyl trimethyl ammonium bromide (CTAB) method^61^. The quality of the DNA was checked by electrophoresis on 0.8% agarose gel and the quantity was determined using a Nano spectrophotometer. A set of 1200 random SSR markers and 225 SNP markers were used to identify polymorphic SSR and SNP (single nucleotide polymorphic) markers between parents, PDK Shriram and Heera. PCR was performed in a 20 μl reaction mixture using 5 pM (pico-mole) of each SSR marker forward and reverses primer, 200 μM of dNTP (dATP, dCTP, dTTP, dGTP), 1U of *Taq* DNA polymerase, 10 mM Tris–HCl (pH = 8.3), 50 mM KCl and 2 mM MgCl2 and 40 ng of genomic DNA. The PCR amplification was carried out in a thermal cycler (Applied Biosystems, USA) with the following parameters: initial denaturation at 94 °C for 3 min followed by 35 cycles of denaturation at 94 °C for 30 s, annealing at 55–65 °C for 1 min and extension at 72 °C for 1.5 min and then a final extension for 10 min at 72 °C. The amplified products were separated on 3–3.5% agarose gels using 1 × TBE buffer, stained with Ethidium bromide (0.5 μg/ml). The gels were visualized under UV radiation and were photographed using Gel-Doc System (Syngene, USA) to detect amplified fragments. All the microsatellite markers used in this study were available in the Gramene database (http://www.gramene.org). 118 polymorphic SSR markers (Supplementary Table S2) were used to genotype 188 RILs along with parents. 225 SNP sequences were downloaded from OryzaSNP@MSU databases (http://rice.plantbiology.msu.edu) and the MassARRAY Assay Design Suite (ADS) software from Agena (www.AgenaCx.com) was used to design assay panels (nine assay panels) for the PCR and iPLEX extension primers for each SNP of interest. These nine assay design panels were used to survey parental polymorphism using the Agena MassArray Analyzer4 (MA4) genotyping system. Depending upon the complexity of the assay, a single multiplex reaction contained 20–33 SNP markers. Two PCR amplifications were carried out. 1^st^ PCR amplification was carried out in a 96-well microtiter plate using 40 ng DNA of each genotype and reagents supplied in the iPLEX reagent kits. PCR conditions like reaction volume or conditions of the PCR program were determined by the kit. After completion of 1st PCR, excess nucleotides were dephosphorylated by using 2 μl of shrimp alkaline phosphatase (SAP) treatment for about 40 min followed by inactivation of SAP enzyme for 5 min at 85 °C. Then 2nd PCR with the iPLEX single base extension reaction in which a mix of oligonucleotide extension primers designed to anneal to the amplified DNA fragments was added together with an extension enzyme and mass-modified dideoxynucleotide terminators. After completion of the 2nd PCR reaction, 30 μl of sterile molecular grade water was added to each well. Then the 96-well microtiter plate was loaded to Agena MassArray Analyzer4 system. The extension products (analytes) were desalted using 13 μl of Clean Resin by transferring to each well of microtiter plate and then 9 nanoliters of reaction PCR products from each well of microtiter plate were loaded onto a SpectroCHIP Array by automated nanodispenser, where they crystalized with a pre-spotted MALDI matrix. The SpectroCHIP Array was then loaded into the main chamber of the Agena MassARRAY Analyzer4 system, where very low pressure is maintained. The UV laser beam was then bombarded precisely on to the each sample (as analyte crystals) present in SpectroCHIP Array. The sample (analyte crystals) was ionized, and the positively charged molecules move from the bottom towards the top (detector) of the chamber. The separation of ionized molecules occurs during movement inside the chamber, which is proportional to the mass of the individual molecule. After each laser bombardment, the detector records the relative time of arrival of each ionized molecule. The lightest ionized molecule arrives first, while the heaviest ionized molecule arrives last at the detector. Typer software automatically generates reports in the form of peaks that identify the SNP alleles (one peak for homozygous while two peaks for heterozygous alleles) in each sample. Twenty-two polymorphic SNP markers (Supplementary Table S3) were identified and subjected to again design assay panel (one panel). SNP genotyping was performed in 188 RILs and parents using the Agena MassArray Analyzer4 genotyping system.

### Construction of linkage map and identification of QTLs

The genotype data on 188 RILs generated by 118 polymorphic SSR and 22 single nucleotide polymorphic (SNP) markers was used for the construction of linkage map using integrated QTL IciMapping, Version 4.1 software^62^. LOD score 3 was used to construct the linkage group. The Kosambi mapping function was used to convert the recombination frequency into genetic distances in centi-Morgan (cM)^63^. The data of linkage map construction was used for QTL identification using the same QTL IciMapping, Version 4.1 software. The inclusive composite interval mapping (ICIM) was used to identify QTLs associated with spikelet fertility percentage, inter-grain space (panicle compactness), and phytohormone ethylene production. The inclusive composite interval mapping (ICIM) software provided more efficient background control via a two-step mapping strategy as compared to composite interval mapping (CIM). This software avoids the possible increase of sampling variance and complicated background marker selection process in CIM^64^. The one-dimensional scanning of the whole genome was carried out with mapping parameters of 1 cM, the probability value for entering variables (PIN) of 0.001 with a threshold LOD score of 3 to identify significant QTLs^65^.

### In silico analysis for identification of the candidate genes associated with QTLs

The genes located within three important QTL regions were searched using NCBI gene bank (https://www.ncbi.nlm.nih.gov/) and rice genome annotation project (RAP-DB) (https://rapdb.dna.affrc.go.jp/). These QTL regions are between Affx93259116 and RM12224 on chromosome 1 containing QTL *qETH1.2*, RM588, and RM20045 on chromosome 6 containing three QTLs *qIGS6.1*, *qIGS6.2* and *qETH6.1*, RM20500, and RM20506 on chromosome 6 containing two QTLs *qSFP6.1* and *qIGS6.3*. The position of genes/loci was obtained from the Gramene database (http://www.gramene.org). The number of genes in QTL regions was listed and candidate genes were selected.

### Expression studies of the candidate genes

Apical and basal spikelets were sampled on 6, 9, and 12 days after anthesis in both parents PDK Shriram and Heera, frozen into liquid nitrogen, and stored at – 80 °C until use. Among RILs, one high spikelet number compact panicle line, 166A, and one lax panicle low spikelet number line, 14A were also considered for the sampling of the spikelets, similar to that of the parents. Total RNA was isolated from the collected samples using TRIZOL reagent (Invitrogen) following the reagent user manual. The quality and quantity of the RNA isolated from the individual samples were checked on Agarose gel and Nanodrop spectrophotometer, respectively. Quanti-Tect Reverse Transcription Kit (Qiagen) was used for conversion of total isolated RNA to cDNA following the protocol outlined in the kit’s manual. The study of the expression of some genes associated with identified QTLs regions were conducted using the cDNAs prepared from the individual samples. Expression of a gene was studied by *q*RT-PCR taking the cDNA as template and SYBR green (Agilent). Primers specific to the gene of interest were designed using Primer Blast software at the NCBI site. The required amount of SYBR green, cDNA template, and the primers for a gene was mixed in a final volume of 20 µl, and PCR was run in Real-Time PCR (Bio-Rad CFX). Rice actin was used as an internal control. The relative level of templates of the individual gene in the apical and basal spikelets was quantified following Pfaffl^66^, and the result was expressed as a fold change in expression in the basal spikelets over apical ones. Seven candidate genes were selected from three QTL regions and used in the expression studies (Supplementary Table S6).

## Supplementary Information

Supplementary Information 1.

Supplementary Information 2.

## Data Availability

Supporting data used in this manuscript available online.
