# Supplementary material for: Identification of novel QTLs for grain fertility and associated traits to decipher poor grain filling of basal spikelets in dense panicle rice
Source: Sci Rep. 2021 Jun 30;11:13617. doi: 10.1038/s41598-021-93134-7 (PMC8245594; doi:10.1038/s41598-021-93134-7)

Supplementary Fig. 1 Frequency distribution of RILs for the traits, spikelet fertility percentage (SFP), inter-grain space(cm)(IGS) and ethylene (ETH) production (pmole/gfwt/ml (air)/hr), derived from PDK Shriram (HGN) and Heera. X-axis shows values of traits and Y-axis shows frequency RILs for the traits, SFP, IGS and ETH in *kharif* 2017 (3a), and *in kharif* 2018 (3b).

Supplementary Fig. 2 Relative expression level in fold of ethylene-responsive genes and *GBSS1* (a) Ethylene responsive transcription factor 8 also named as ethylene responsive factor 3 (*ERF3*), (b) AP2-like ethylene-responsive transcription factor (*AP2-ERF*), (c) Ethylene-responsive element binding protein (EREBP) transcription factor, and (d) Granule bound starch synthase 1 (*GBSS1*) on 6th day, 9th day and 12th day after anthesis in the basal over apical spikelets of compact panicled PDK Shriram (HGN), RIL-166A and lax-panicled Heera, RIL-14A, determined by qRT-PCR. Rice actin was taken as internal reference. Data represent mean (±SD) using three biological replicates.

Supplementary Fig. 3 Relative expression level in fold of (a) E3 ubiquitin-protein ligase *GW2*, (b) serine carboxypeptidaseII-2, and (c) LRR receptor-like serine/ threonine-protein kinase ERL1 on 6th day, 9th day and 12th day after anthesis in the basal over apical spikelets of compact panicled PDK Shriram (HGN), RIL-166A and lax-panicled Heera, RIL-14A, determined by qRT-PCR. Rice actin was taken as internal reference. Data represent mean (±SD) of three biological replicates.

Supplementary Fig. 1.

a

Kharif season 2017

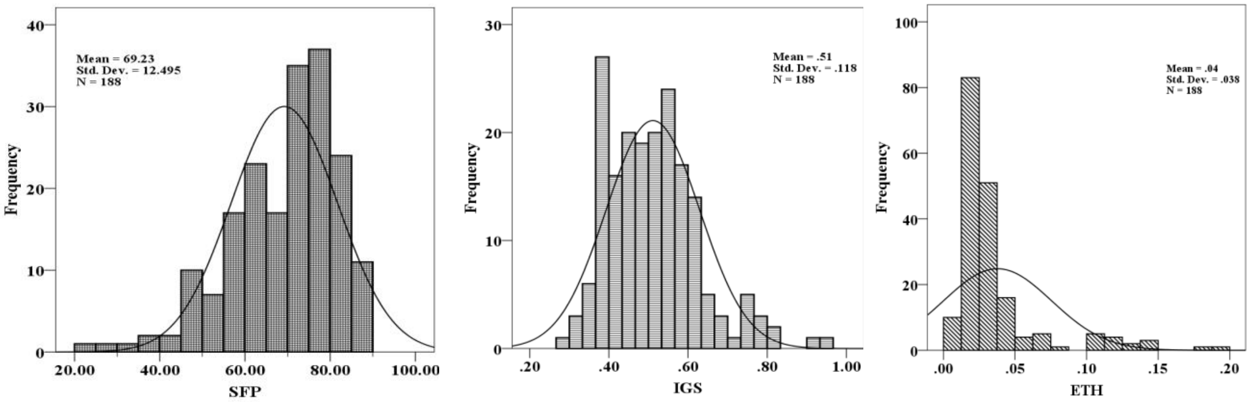

b

Kharif Season 2018

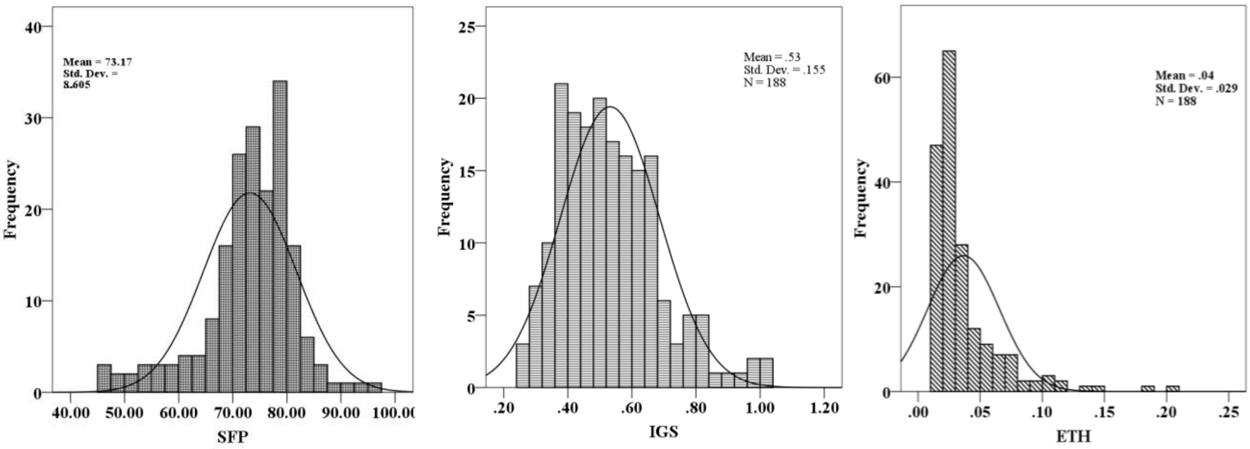

Supplementary Fig. 2.

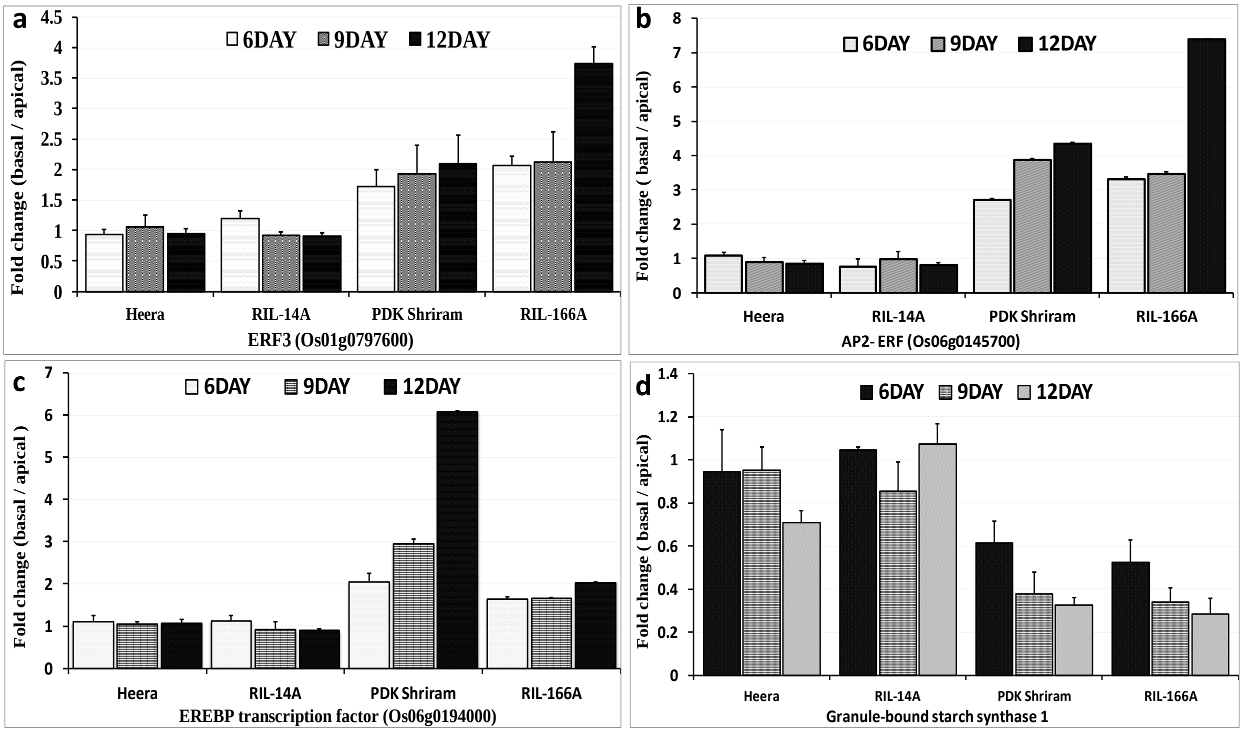

Supplementary Fig. 3.

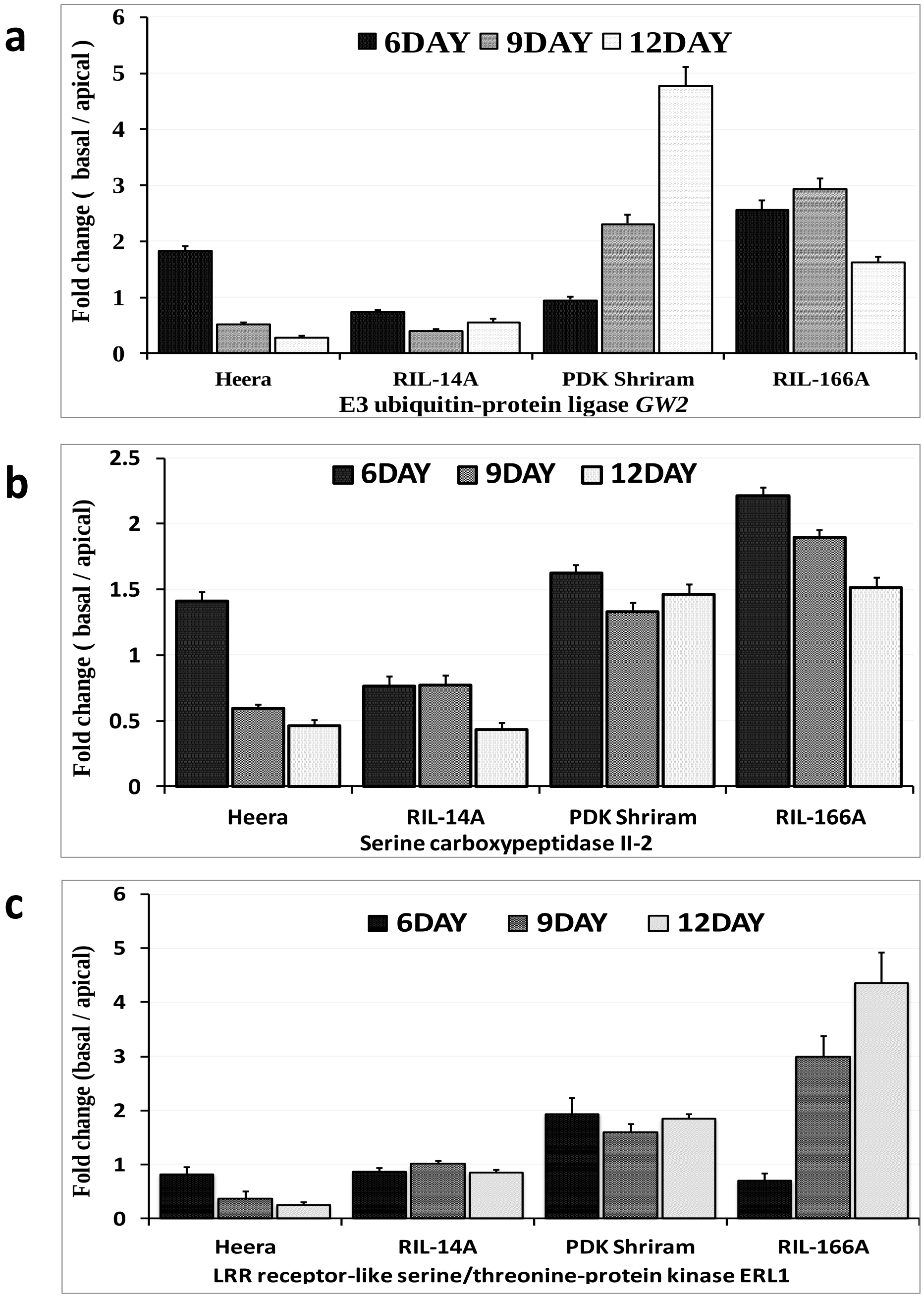

Supplement: Supplementary file 1 — Supplementary Information 1. [file 41598_2021_93134_MOESM1_ESM.pdf]
